# Maternal postnatal depression and offspring depression at age 24 years in a UK-birth cohort: the mediating role of maternal nurturing behaviours concerning feeding, crying and sleeping

**DOI:** 10.12688/wellcomeopenres.17006.2

**Published:** 2022-10-19

**Authors:** Iryna Culpin, Gemma Hammerton, Marc H. Bornstein, Jon Heron, Jonathan Evans, Tim Cadman, Hannah M. Sallis, Kate Tilling, Alan Stein, Alex S.F. Kwong, Rebecca M. Pearson

**Affiliations:** 1 Centre for Academic Mental Health, Population Health Sciences, Bristol Medical School, University of Bristol, Bristol, BS82BN, UK; 2 MRC Integrative Epidemiology Unit, University of Bristol, Bristol, BS82BN, UK; 3 Eunice Kennedy Shriver National Institute of Child Health and Human Development, National Institute of Child Health and Human Development, Bethesda, MD, USA; 4 Institute for Fiscal Studies, Institute for Fiscal Studies, London, UK; 5 NIHR Biomedical Research Centre, University of Bristol, Bristol, UK; 6 School of Psychological Science, University of Bristol, Bristol, UK; 7 Department of Psychiatry, Medical Sciences Division, University of Oxford, Oxford, UK; 8 MRC/Wits Rural Public Health and Health Transitions Research Unit (Agincourt), School of Public Health, Faculty of Health Sciences, University of the Witwatersrand, Johannesburg, South Africa; 9 Division of Psychiatry, Centre for Clinical Brain Sciences, University of Edinburgh, Edinburgh, UK

**Keywords:** ALSPAC, maternal postnatal depression, offspring depression; maternal early nurturing parenting behaviours, population-based study.

## Abstract

**Background:** Maternal postnatal depression (PND) is a risk factor for offspring depression in adulthood. However, few longitudinal studies have examined the role of maternal nurturing parenting behaviours in the association between maternal PND and offspring depression in adulthood.

**Methods:** We examined pathways from maternal PND measured using self-reported Edinburgh Postnatal Depression Scale at 8 weeks to offspring ICD-10 depression diagnosed using the Clinical Interview Schedule-Revised computerised assessment at 24 years through maternal-reported nurturing behaviours concerning feeding, sleeping and crying measured from pregnancy to age 3 years 6 months in 5,881 members of the UK-based birth cohort study, the Avon Longitudinal Study of Parents and Children.

**Results: **The fully adjusted model revealed an indirect effect from PND to adult offspring depression through the combination of all parenting factors (probit regression coefficient [ *B*]=0.038, 95% confidence interval [CI] 0.005, 0.071); however, there was no evidence of a direct effect from early maternal PND to offspring depression once the indirect effect via parenting factors was accounted for ( *B*=0.009, 95%CI -0.075, 0.093). Specificity analyses revealed indirect effects through maternal worries about feeding ( *B*=0.019, 95%CI 0.003, 0.035, p=0.010) and maternal perceptions and responses to crying ( *B*=0.018, 95%CI 0.004, 0.032, p=0.012).

**Conclusions: **The adverse impact of maternal PND on offspring depression in early adulthood was explained by maternal nurturing behaviours concerning feeding, crying and sleeping in early childhood. Residual confounding and measurement error likely limit reliable conclusions. If found causal, interventions providing support to reduce worries around maternal nurturing behaviours and treating depression could reduce adverse outcomes in adult offspring of depressed mothers.

## Introduction

Substantial research supports the association between maternal postnatal depression (PND) and increased risk of later psychological problems in offspring ( Stein *et al*., 2014). However, few large prospective longitudinal studies have examined whether these adverse effects persist into adulthood beyond 18–20 years of age ( Weissman *et al*., 2016). Furthermore, the offspring outcomes associated with maternal PND are heterogenous and reported effect sizes are small to moderate ( Stein *et al*., 2014). Thus, it is important to elucidate the long-term effects of maternal PND on offspring mental health beyond adolescence.

In addition, few studies have examined putative mediating factors that underly associations between parental and offspring depression. Insights into possible mediators would be crucial to identify children at greater risk and to develop targeted interventions to reduce adverse outcomes in offspring of depressed mothers. A substantial body of evidence suggests that an important potential mediator is the quality of parenting ( Sanger *et al*., 2015; Stein *et al*., 2014). Specifically, PND disrupts maternal sensitivity ( Murray *et al.*, 2010) and is associated with less engaged parenting ( Lovejoy *et al*., 2000), which is, in turn, associated with adverse offspring outcomes, including mental health problems ( Bornstein, 2015).

Numerous studies have identified aspects of parenting that reflect affective, cognitive and physical symptoms of maternal PND ( Lovejoy *et al*., 2000), including lower warmth, sensitivity and responsiveness ( Bornstein, 2015). However, much less attention has been paid to the importance of everyday basic maternal nurturing activities that are essential to infant care, or their potential explanatory role in associations between maternal and offspring depression. Caring for infants, who are fully dependent on their parents, require high levels of caregiving involvement normally focused on meeting infants’ basic needs for feeding, responding to their crying, and managing their sleeping routines. Importantly, these basic needs are impossible to ignore, unpredictable and stressful, and thus may be particularly challenging for parents with depression. Approximately 20–25% of parents report problems feeding infants in the first 2 years ( Lindberg *et al.*, 1991) with food refusal and fussiness being common features ( Young & Drewett, 2000). Infant crying peaks in the first 3 months, including an increase in prolonged night-time crying ( Walker & Menahem, 1994). Initiating and maintaining child sleep is a persistent issue during the first years, challenging parents with long evening sleep rituals, waking at night and coming into the parental bed ( Stoleru *et al*., 1997; Wolke, 2003). Although feeding, crying and sleeping patterns in young children are highly variable, they nevertheless are universal features and challenges of early child development, thus calling for a range of parenting managements strategies. 

Maternal PND is associated with child feeding, including food refusal and fussy eating ( Coulthard & Harris, 2003), as well as maternal feeding behaviours (i.e., feeding style and practices), with depressed mothers resorting to more physical and verbal pressures and offering more incentives to encourage their children to eat ( Haycraft *et al.*, 2013). PND has also been associated with more crying per day ( Milgrom *et al.*, 1995), longer episodes of crying/fussing and increased crying bout frequency ( Miller *et al.*, 1993), as well as reduced sensitivity and responsiveness to infant crying (e.g., feeding, rocking and touching; Esposito *et al*., 2017). Similarly, ample evidence has established a link between maternal PND and sleep difficulties in infants and young children ( Karraker & Young, 2007), including increased frequency of child awakenings ( Warren *et al*., 2006). Sleeping difficulties may have an impact on how mothers approach bedtime routines, including regularity, sleep ecology (i.e., sleep location) and night waking behaviours.

Parenting is part of a transactional dynamic ( Bornstein, 2009; Mills-Koonce *et al*., 2007) with children shaping parenting behaviour ( Avinun & Knafo, 2014). Children who are more difficult to look after due to fussy eating, frequent crying, and poor sleep routines may evoke mood changes in caregivers and trigger more reactive and less consistent parenting ( Stein *et al*., 2014). These so-called ‘evocative’ child effects are important to account for as findings regarding parental influences on child outcomes likely reflect child influences ( Avinun & Knafo, 2014). This dynamic is of particular importance in the context of maternal and offspring depression, which share common genetic liabilities ( Knafo & Jaffee, 2013). 

Although quality of parenting is important for offspring growth and development ( Smith, 2010), little is known regarding the role that maternal basic nurturing parenting behaviours concerning feeding, crying and sleeping (henceforth referred to as ‘maternal nurturing behaviours’) play in the association between maternal and offspring depression. In the current study we address this gap by examining the impact of maternal PND on such nurturing parenting behaviours and estimating the extent to which the association between maternal PND and offspring depression at age 24 years is explained by early maternal nurturing behaviours using data from a large UK-based birth cohort study, the Avon Longitudinal Study of Parents and Children (ALSPAC). Quantifying this association may inform preventative and intervention programmes given that parenting behaviours are modifiable ( Kaminski *et al*., 2008). The richness of the ALSPAC data provides a unique opportunity to account for a number of factors associated with both maternal and offspring depression, including child polygenic score for neuroticism that may indicate genetic confounding. Our specific research questions were:

1. Is maternal PND associated with offspring depression at age 24 years?
2. Is any observed association between maternal PND and offspring depression mediated by maternal nurturing behaviours?

## Methods

### Study cohort

The sample comprised participants from the ALSPAC cohort. During Phase I enrolment, 14,541 pregnant mothers residing in the former Avon Health Authority in the south-west of England with expected dates of delivery between 1 April 1991 and 31 December 1992 were recruited. The total sample size is 15,454 pregnancies, of which 14,901 were alive at 1 year of age. Our sample comprised 12,986 mothers with at least one parenting item. Ethical approval and informed consent for data collection were obtained from the ALSPAC Ethics and Law Committee and the Local Research Ethics Committees. Details of specific ethics approvals are available on the website. Information about ALSPAC is available on the website, including a searchable data dictionary. Further details on the cohort profile, representativeness and phases of recruitment are described in three cohort-profile papers ( Boyd *et al*., 2013; Fraser *et al*., 2013; Northstone *et al*., 2019). 

### Measures

***Exposure: maternal postnatal depression.*** Symptoms of maternal PND were measured using the Edinburgh Postnatal Depression Scale (EPDS; Cox *et al.*, 1987), a 10-item self-reported depression questionnaire validated for use during the perinatal period and posted to mothers at 8 weeks postnatally. Confirmatory Factor Analysis (CFA) was used to derive a normally distributed latent trait based on 10-EPDS ordinal response items (see Methods S1, Extended data ( Culpin *et al*., 2021)).

***Outcome: offspring depression.*** Offspring depression was assessed using the computerised version of the Clinical Interview Schedule-Revised (CIS-R; Lewis *et al*., 1992), a fully structured psychiatric interview widely used in the community samples. Participants were invited to attend a research clinic at age 24 years and complete computerised assessment to identify individuals with an ICD-10 diagnosis of depression ( *versus* no diagnosis).

***Mediators: maternal nurturing behaviours.*** Full details pertaining to item selection and development of factors encapsulating maternal nurturing behaviours concerning feeding, crying and sleeping are presented in Methods S1 (Extended data ( Culpin *et al*., 2021)). We extracted items from maternal self-reported questionnaires administered from birth to age 3 years 6 months (8 occasions) capturing maternal feeding style and practices, perceptions and responses to crying, and strategies to regulate bedtime routine and sleep ecology. These items were entered into separate CFA models per each dimension.

***Potential confounders: child polygenic score for neuroticism, socioeconomic, parental and family characteristics.*** Analyses were adjusted for child polygenic score (PGS) for neuroticism to account for possible genetic confounding ( Stein *et al*., 2014). Neuroticism PGS has been found to be a robust predictor of a number of psychiatric disorders, including major depressive disorder in population-based samples ( Docherty *et al*., 2016; Luciano *et al*., 2018). Genotyped data were available for 8,237 children in the ALSPAC (full details in Methods S1, Extended data ( Culpin *et al*., 2021)). In addition, disadvantaged socioeconomic status and marital conflict are strong risk factors for maternal PND ( Leigh & Milgrom, 2008) and less optimal parenting practices ( Dix & Moed, 2019; Hoff & Laursen, 2019). Thus, we adjusted for a range of potential confounding factors collected prospectively from maternal questionnaires during the antenatal period: highest maternal educational attainment (minimal education or none/compulsory secondary level (up to age 16 years; O-Level) *versus* non-compulsory secondary level (up to age 18 years; A-Level)/university level education), maternal age in years, family size (1 child *versus* ≥1 child), early parenthood (dichotomised as ≤19 years *versus* ≥ 20 years), perceived affordability of the cost of living (yes *versus* no), and parental conflict/aggression derived from questions asking how the mother and her current partner behaved towards each other (yes *versus* no). 

### Statistical analysis

***Analyses plan.*** We first derived latent factor models that encapsulated maternal PND (8 weeks) and maternal nurturing behaviours in infancy (0-3 years) concerning sleeping, feeding and crying using Confirmatory Factor Analyses (CFA). Second, we estimated the extent to which the association between maternal PND (8 weeks) and offspring (24 years) depression (total effect) is mediated by specific factors related to maternal nurturing parenting behaviours (0-3 years; indirect effects). We used Structural Equation Modelling (SEM) in Mplus v.8.2 ( Muthen & Muthen, 2015) with latent constructs to estimate unadjusted models and models incrementally adjusted for child, maternal and socioeconomic characteristics. Third, we conducted sensitivity analyses to examine the impact of missing data on our findings. We re-ran our analyses (with maternal depression at 8 months as a sum-score) using multiple imputed datasets and compared the results to complete case analyses (also using maternal depression as a sum-score).

**Figure 1.**
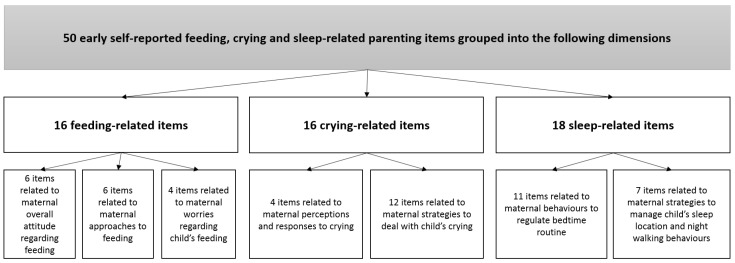
Flow chart depicting items includes into the final Confirmatory Factor Analysis (CFA) model.

***Latent factor model.*** The hypothesised latent factor model is represented in Figure S1 (Extended data ( Culpin *et al*., 2021)). Full details of latent factor model derivation, including the flow chart of items included into the CFA and derived factors and factor loadings are presented in Figure 1 and Table 1. In summary, items that were both theoretically relevant and showed standardised loadings (>0.15) on the relevant parenting dimension were included in a combined model using CFA with a robust Weighted Least Square (WLSMV) estimator to model categorical and continuous data ( Brown & Moore, 2012). Root Mean Square Error of Approximation (RMSEA; >0.06), Comparative Fit Index (CFI) and Tucker-Lewis Index (TLI; >0.95) were used to evaluate model fit ( Hu & Bentler, 1998). The chi-square test of overall fit is prone to model misspecification when sample size is large ( Lomax & Schumacker, 2004); thus, we gave preference to relative fit indices.

**Table 1.** Derived factors, items, standardised factor loadings and fit indices for the measurement model.

Age/Factor	Items	Factor loading	S.E.
**Feeding style**			
8 months	Regular feed and sleep are important	0.277	0.030
1 year 3 months	Child fed on demand	0.583	0.020
1 year 3 months	Child wants to feed herself/himself	0.167	0.020
1 year 3 months	Child allowed to feed herself/himself	0.072	0.020
1 year 9 months	Child should eat whenever	0.367	0.017
3 years 6 months	Choices mum allows about food	0.338	0.019
**Feeding practices**			
1 year 3 months	Main meal same as mother	0.233	0.016
1 year 3 months	Main meal different from mother	0.261	0.014
1 year 6 months	Frequency child refuses food mother makes	0.696	0.011
1 year 6 months	Child is given other meal when dislikes main meal	0.649	0.013
1 year 6 months	Child has dessert when refuses main meal	0.619	0.013
1 year 6 months	Child must eat something before dessert	0.475	0.014
**Worries about feeding**			
1 year 3 months	Mother is worried child is not eating enough	0.754	0.009
1 year 3 months	Mother is worried child is refusing food	0.915	0.007
1 year 3 months	Mother is worried child is choosy food	0.871	0.007
1 year 3 months	Mother worried about lack of feeding routine	0.770	0.010
**Perceptions and responses to crying**			
4 weeks	Mother picks child up in reaction to crying	0.758	0.020
8 months	Mother cannot bear child crying	0.311	0.018
6 months	Mother picks child up in reaction to crying	0.694	0.019
1 year 9 months	Mother feels unbearable when child cries	0.311	0.018
**Strategies to manage crying**			
1 year 6 months	Mother gives fruit to stop crying	0.394	0.014
1 year 6 months	Mother gives breast milk to stop crying	0.287	0.032
1 year 6 months	Mother gives milk drink to stop crying	0.342	0.015
1 year 6 months	Mother gives other drink to stop crying	0.420	0.014
1 year 6 months	Mother gives other food to stop crying	0.147	0.017
3 years 6 months	Mother gives sweets to stop crying	0.727	0.009
3 years 6 months	Mother gives chocolate to stop crying	0.685	0.010
3 years 6 months	Mother gives crisps to stop crying	0.850	0.007
3 years 6 months	Mother gives fruit to stop crying	0.865	0.007
3 years 6 months	Mother gives milk to stop crying	0.663	0.010
3 years 6 months	Mother gives drink to stop crying	0.821	0.008
3 years 6 months	Mother gives other drink to stop crying	0.396	0.016
**Regularity of bedtime routine**			
1 year 6 months	Child incompliant with mother about bed routine	0.808	0.005
1 year 6 months	Frequency child stays up after bedtime	0.828	0.005
1 year 6 months	Frequency child sleeps before put to bed	0.885	0.004
1 year 6 months	Frequency child made to go to bed	0.383	0.012
1 year 6 months	Frequency child is played with/read before bed	0.612	0.008
1 year 6 months	Frequency child is cuddled until she/he falls asleep	0.891	0.004
1 year 6 months	Frequency child has bottle before bed	0.720	0.007
2 years 6 months	Child has regular sleep routine	0.489	0.016
2 years 6 months	Child sleep pattern evaluation	0.427	0.011
2 years 6 months	Child refuses to go to bed	0.500	0.011
2 years 6 months	Child wakes up early	0.172	0.013
**Sleep ecology**			
1 year 6 months	Room child put down to sleep at night	0.762	0.008
1 year 6 months	Room child wakes up in the morning	0.829	0.008
1 year 6 months	Child shares bed when put down to sleep	0.835	0.011
1 year 6 months	Child shares bed when wakes up in the morning	0.849	0.007
2 years 6 months	Child sleeping place at night	0.783	0.007
2 years 6 months	Child waking place in the morning	0.732	0.009
2 years 6 months	People child sleeps with at night	0.845	0.010
**Latent factor model fit indices**			
Free parameters		267	
Root Mean Square Error of Approximation (RMSEA)		0.033 (95%CI 0.032 to 0.033)	
Comparative Fit Index (CFI)		0.926	
Tucker-Lewis Index (TLI)		0.918	

***Direct and mediated effects.*** Multifaceted constructs, such as different aspects of parenting, are challenging and important in mediation as each specific factor may relate differentially to the outcome ( Gonzalez & MacKinnon, 2018). Full details of the mediation model to examine direct and indirect (mediated) effects are presented in Method S1 (Extended data ( Culpin *et al*., 2021)). In summary, we examined the extent to which the association between maternal postnatal (8 weeks) and offspring (24 years) depression (total effect) is mediated by specific factors related to maternal nurturing behaviours (0–3 years; indirect effects). Analyses of longitudinal mediation models were restricted to those with complete data on the child neuroticism PGS and antenatal confounders (n=5,881). We used Structural Equation Modelling (SEM) in M *plus* v.8.2 ( Muthén & Muthén, 2015) with latent constructs of maternal depression and parenting to estimate unadjusted (Model ^a^: including exposure, outcome and mediator only) and incrementally adjusted models (Model ^a+b^: adjusted for child neuroticism PGS; Model ^a+b+c^: further adjusted for socioeconomic and maternal characteristics; Model ^a+b+c+d^: further adjusted for parental conflict; Table 5). Results from path analyses with binary outcome (offspring diagnosis of depression), including indirect effects, are presented as probit regression coefficients (hereafter referred to as *B,* details in Methods S1, Extended data ( Culpin *et al*., 2021)). Probit coeffecients represent the predicted probability of the outcome (offspring depression) for a 1 unit increase in the exposure (maternal PND). We conducted sensitivity analyses with early diagnosis of offspring depression (CIS-R; Lewis *et al*., 1992) and depressive symptoms (MFQ; Messer *et al*., 1995) at 18 years as outcomes (Results S1, Extended data ( Culpin *et al*., 2021)).

***Missing data: multiple imputation.*** We conducted sensitivity analyses to examine the impact of missing data on our findings. Full description of the imputation method is presented in Method S1 (Extended data ( Culpin *et al*., 2021)).

## Results

### Characteristics of the cohort by the exposure status

The characteristics of our study sample and prevalence of offspring depression at age 24 years by the presence of maternal PND are presented in Table 2. In summary, mothers who became parents at an earlier age, had more children, and reported conflictual relationship with the partner and difficulties with affording the cost of living were more likely to report depressive symptoms than those who became parents later in life, had one child, and did not experience financial difficulties. Mothers with higher levels of education (A-Level/university degree) were more likely to report depressive symptoms than mothers with lower levels of educational attainment, whilst younger mothers were no more likely to report depressive symptoms than older mothers. There was some evidence that offspring of depressed mothers were more likely to have been diagnosed with depression at age 24 years than those whose mothers did not experience depression during the early postnatal period. 

**Table 2.** Characteristics of the cohort and prevalence of offspring depression at age 24 years old by the exposure status (maternal postnatal depression (PND) at 8 months).

Exposure status: N (%)	Maternal postnatal depression (8 months)	
	No 10,388 (89.9)	Yes 1,171 (10.1)
	*n (%)*	*n (%)*
*Maternal educational attainment*		
A-Level/Degree	5,721 (89.8)	653 (10.2)
Minimal education/none/O-Level	3,720 (91.4)	350 (8.6)
Chi2, p-value		7.74, 0.005
*Early parenthood*		
≥ 20 years	9,741 (90.5)	1,022 (9.5)
≤19 years	647 (81.3)	149 (18.7)
Chi2, p-value		69.25, <0.001
*Parental conflict/aggression*		
No	5,648 (93.2)	409 (6.8)
Yes	3,978 (86.4)	624 (13.6)
Chi2, p-value		138.44, <0.001
*Family size*		
1 child	8,068 (90.6)	835 (9.4)
≥1 child	1,893 (87.4)	272 (12.6)
Chi2, p-value		19.62, <0.001
*Affordability*		
No	7,489 (92.9)	571 (7.1)
Yes	2,200 (81.8)	491 (18.2)
Chi2, p-value		282.32, <0.001
*Maternal age, mean (SD)*	*27.8 (4.7)*	*27.6 (5.0)*
ANOVA, p-value		1.34, 0.180
*Offspring diagnosis of depression* *at 24 years old*		
No	2,735 (91.8)	245 (8.2)
Yes	320 (88.6)	41 (11.4)
Chi2, p		4.05, 0.044

*Note*: p-values based on Pearson’s Chi-square (Chi ^2^) test of association between maternal postnatal depression and categorical variables, and ANOVA for differences in means for continuous variables; sample sizes vary due to the differences in data availability on socioeconomic, parental and familial characteristics.

### Associations between maternal nurturing behaviours

Maternal feeding, crying and sleep-related parenting behaviours were relatively highly correlated ( Table 3). In summary, maternal feeding style more concordant with authoritative parenting (i.e., higher levels of maternal responsiveness and appreciation of feeding routine) were associated with less maternal worry regarding child’s feeding, as well as less pressured and restricting feeding behaviour. In contrast, feeding style less concordant with authoritative parenting (i.e., more pressured and restricting maternal feeding behaviour) was associated with more maternal worry regarding child’s feeding, less optimal and responsive strategies to manage and respond to child’s crying, less adherence to bedtime routine and worse sleep ecology. Maternal worries about child’s feeding were associated with less responsive and sensitive maternal responses to crying and less optimal strategies to console the child. Maternal adherence to regular sleep routine was associated with more consistent and adaptive sleep ecology (i.e., child was more likely to wake up in the same place where she was put to bed). More responsive and sensitive maternal responses to child’s crying were associated with more optimal strategies to manage child’s crying (e.g., giving milk rather than chocolate), more maternal adherence to regular sleep routine, less reactive responses to child’s non-compliance with bedtime routine and better sleep ecology. 

**Table 3.** Associations between final maternal nurturing behaviours concerning feeding, crying and sleeping with each other ^1^.

Parenting factor		Point estimate (β)	S.E.	p-value
*Feeding style*	Feeding practices	-0.170	0.018	≤0.001
	Worries about feeding	0.194	0.018	≤0.001
	Perceptions and responses to crying	-0.377	0.021	≤0.001
	Strategies to manage crying	-0.198	0.020	≤0.001
	Regularity of bedtime routine	-0.274	0.018	≤0.001
	Sleep ecology	-0.371	0.018	≤0.001
*Feeding practices*	Worries about feeding	-0.460	0.011	≤0.001
	Perceptions and responses to crying	-0.377	0.021	≤0.001
	Strategies to manage crying	-0.198	0.020	≤0.001
	Regularity of bedtime routine	-0.274	0.018	≤0.001
	Sleep ecology	-0.371	0.018	≤0.001
*Worries about feeding*	Perceptions and responses to crying	-0.155	0.015	≤0.001
	Strategies to manage crying	-0.128	0.013	≤0.001
	Regularity of bedtime routine	-0.219	0.012	≤0.001
	Sleep ecology	-0.077	0.013	≤0.001
*Perceptions and responses to crying*	Strategies to manage crying	0.042	0.042	0.010
	Regularity of bedtime routine	0.151	0.014	≤0.001
	Sleep ecology	0.123	0.015	≤0.001
*Strategies to manage crying*	Regularity of bedtime routine	0.222	0.013	≤0.001
	Sleep ecology	0.076	0.014	≤0.001
*Regularity of bedtime routine*	Sleep ecology	0.333	0.011	≤0.001

Note: ^1^ Effect size are regression coefficients ( *β* unstandardised)

### Associations between maternal postnatal depression, nurturing behaviours and offspring depression

The associations between maternal nurturing behaviours and offspring depression are presented in Table 4.

**Table 4.** Associations between maternal postnatal depression (8 weeks), offspring depression (24 years) and final maternal nurturing behaviours concerning feeding, crying and sleeping (n=5,881).

Parenting factor	Maternal postnatal depression (8 weeks) ^a^		Offspring depression (24 years)	
	Fully adjusted model estimates ^b^ (n=5,881)			
	*Β* [95% CI]	P-value	*Β* [95% CI]	P-value
*Feeding style*	0.059 [0.006. 0.112]	0.030	0.059 [-0.074, 0.192]	0.384
*Feeding practices*	-0.121 [-0.158, -0.084]	≤0.001	-0.018 [-0.110, 0.074]	0.708
*Worries about feeding*	0.172 [0.137, 0.210]	≤0.001	0.113 [0.031, 0.195]	0.008
*Perceptions and responses to crying*	-0.132 [-0.171, -0.093]	≤0.001	-0.135 [-0.233, -0.037]	0.006
*Strategies to manage crying*	-0.083 [-0.120, -0.046]	≤0.001	0.039 [-0.045, 0.123]	0.363
*Regularity of bedtime routine*	-0.080 [-0.115, -0.045]	≤0.001	0.022 [-0.068, 0.112]	0.631
*Sleep ecology*	0.010 [-0.025, 0.045]	0.809	-0.032 [-0.132, 0.068]	0.531

Note: ^a^ Maternal postnatal depression (8 weeks) modelled as a latent factor using Confirmatory Factor Analyses (CFA); ^b^ Effect size are regression coefficients ( *β* unstandardised) in the fully adjusted models: adjusted for child PGS, socioeconomic (maternal educational attainment, family size), maternal (age, early parenthood) characteristics, and parental conflict. PGS: Polygenic Score for Neuroticism.

With the exception of sleep ecology, maternal early postnatal depression was strongly associated with feeding, crying and sleep-related parenting factors in the fully adjusted model controlling for child neuroticism PGS and a range of socioeconomic, parental, and family characteristics. We found evidence that although mothers who experienced postnatal depression seemed to appreciate feeding routine, they may do so in the context of more pressured and restricting feeding behaviours. In addition, they were less likely to appreciate and adhere to regular sleep routine. Maternal postnatal depression was also strongly predictive of more maternal worry regarding child’s feeding and less sensitive maternal perceptions and responses to child’s crying, as well as less optimal strategies to manage crying.

In the fully adjusted model, there was no evidence of an association between maternal feeding and bedtime practices, strategies to manage child’s crying and sleep location and child’s night walking behaviours and offspring depression at age 24 years. However, there was evidence that offspring of mothers, who reported less sensitive perceptions and responses to child’s crying and more worries about child’s feeding, were at higher risk of being diagnosed with depression at age 24 years.

### Factors encapsulating maternal nurturing behaviours

A model using CFA to fit latent factors capturing maternal nurturing behaviours suggested an adequate measurement model fit (RMSEA: 0.033, 95%CI 0.032 to 0.033; CFI/TLI: 0.926/0.918; Table 1 supporting further tests of structural paths (direct and mediated effects). Seven factors representing maternal nurturing behaviours concerning feeding, crying and sleeping were derived (full details in Results S1, Extended data ( Culpin *et al*., 2021)). 

*Factor 1 Feeding style*: maternal overall attitude regarding feeding, with higher factor scores representing maternal feeding style more concordant with authoritative parenting (i.e., higher levels of maternal responsiveness and appreciation of feeding routine).

*Factor 2 Feeding practices*: maternal approaches to feeding, with higher factor scores representing less pressured and restricting feeding behaviour (e.g., child is given a different meal when dislikes main meal).

*Factor 3 Worries about feeding*: maternal worries regarding child feeding, with higher factor scores representing higher levels of maternal worry regarding child’s feeding, so unlike other factors higher scores are predicted to confer greater risk.

*Factor 4 Perceptions of and responses to crying*: maternal feelings and behaviours in response to child crying, with higher factor scores representing more sensitive maternal responses to child’s crying. 

*Factor 5 Strategies to manage crying*: maternal strategies to deal with child crying, with higher factor scores representing more optimal responses to child crying (e.g., giving milk rather than chocolate to stop crying).

*Factor 6 Regularity of bedtime routine*: maternal behaviours to regulate bedtime routine, with higher factor scores representing more maternal adherence to regular sleep routine and less reactive response to child non-compliance with bedtime routines.

*Factor 7 Sleep ecology*: maternal strategies to manage child sleep location and night waking behaviours, with higher factor scores representing more consistent and adaptive maternal responses to sleep location and child night waking behaviours (e.g., child sleeping in own bed and in own room, child put down to sleep at night and waking up in same room).

### Direct and mediated effects

We estimated unadjusted and adjusted structural mediation models to examine the direct effect of maternal PND on offspring depression and the mediated effects through specific maternal nurturing behaviours whilst accounting for a range of confounders (Figure S2, Extended data ( Culpin *et al.,* 2021)). Of 3,567 young adults with data on depression diagnosis at age 24 years, 384 had depression (10.8%, 95%CI 0.09, 0.12). Females (13.1%, 95%CI 0.12, 0.14) had higher prevalence of depression than males (6.9%, 95%CI 0.06, 0.08). There was some evidence of total indirect effect from early maternal PND to offspring depression at age 24 years through the combination of all specific maternal nurturing behaviours in the unadjusted (probit regression coefficient [ *B*] =0.023, 95%CI -0.002, 0.048, p=0.072) and fully adjusted models ( *B* =0.038, 95%CI 0.005, 0.071, p=0.027), although the 95% CIs were wide. There was some evidence of total effect in the unadjusted model ( *B*=0.082, 95%CI 0.004, 0.160, p=0.040), which was substantially attenuated in the fully adjusted model ( *B*=0.046, 95%CI -0.030, 0.122, p=0.240). In line with Loeys *et al*. (2015), these analyses may be more powered to detect indirect effect than total effect given that the indirect effect is composed of two proximal effects, whereas the total effect is more distal.

There was no evidence of a direct effect from maternal PND to offspring depression at age 24 years in the unadjusted ( *B* =0.059, 95%CI -0.023, 0.141, p=0.156) and fully adjusted ( *B* =0.009, 95%CI -0.075, 0.093, p=0.839) models once the indirect effect via maternal nurturing behaviours was accounted for ( Table 5).

**Table 5.** Estimates of the direct and mediated effects in the specific factors mediator model unadjusted and adjusted for child PGS and antenatal confounders in complete sample (n=5,881; exposure: maternal depression modelled as a latent factor).

	Model estimates (n=5,881)							
Effect Size ^1^	Unadjusted model ^a^		Adjusted model ^a+b^		Adjusted model ^a+b+c^		Adjusted model ^a+b+c+d^	
	*Β* [95% CI]	P- value	*Β* [95% CI]	P- value	*Β* [95% CI]	P- value	*Β* [95% CI]	P- value
*1. Total effect* Early maternal postnatal depression on offspring depression	0.082 [0.004, 0.160]	0.040	0.083 [0.005, 0.161]	0.037	0.051 [-0.027, 0.129]	0.199	0.046 [-0.030, 0.122]	0.240
*2. Direct effect* Early maternal postnatal depression on offspring depression, accounting for all specific parenting factors	0.059 [-0.023, 0.141]	0.156	0.061 [-0.021, 0.143]	0.147	0.019 [-0.065, 0.103]	0.669	0.009 [-0.075, 0.093]	0.839
*3. Total indirect* Early maternal postnatal depression on offspring depression, through all specific parenting factors	0.023 [-0.002, 0.048]	0.072	0.022 [-0.003, 0.047]	0.075	0.033 [0.002, 0.064]	0.037	0.038 [0.005, 0.071]	0.027
*4. Specific indirect effects* Early maternal postnatal depression on offspring depression, through:								
*Feeding style*	0.002 [-0.004, 0.008]	0.489	0.002 [-0.004, 0.008]	0.486	0.003 [-0.075, 0.081]	0.452	0.003 [-0.005, 0.011]	0.429
*Feeding practices*	0.001 [-0.007, 0.009]	0.902	0.001 [-0.007, 0.009]	0.917	0.002 [-0.010, 0.014]	0.716	0.002 [-0.010, 0.014]	0.709
*Worries about feeding*	0.019 [0.005, 0.033]	0.008	0.019 [0.005, 0.033]	0.008	0.020 [0.004, 0.036]	0.010	0.019 [0.003, 0.035]	0.010
*Perceptions and responses to * *crying *	0.004 [-0.002, 0.010]	0.181	0.004 [-0.002, 0.010]	0.170	0.013 [0.001, 0.0245]	0.017	0.018 [0.004, 0.032]	0.012
*Strategies to manage crying*	-0.003 [-0.011, 0.005]	0.416	-0.003 [-0.011, 0.005]	0.406	-0.003 [-0.011, 0.005]	0.395	-0.003 [-0.011, 0.005]	0.372
*Regularity of bedtime routine*	-0.001 [-0.005, 0.003]	0.790	-0.001 [-0.005, 0.003]	0.761	-0.002 [-0.008, 0.004]	0.632	-0.002 [-0.010, 0.006]	0.633
*Sleep ecology*	0.001 [-0.001, -0.003]	0.507	0.001 [-0.003, 0.001]	0.497	0.001 [-0.002, 0.003]	0.610	0.001 [-0.001, 0.003]	0.667

*Note:* Analyses restricted to individuals with complete data on child PGS and antenatal confounders; ^1^ effect size are unadjusted and adjusted probit regression coefficients ( *B* unstandardised); ^a^ unadjusted model; ^a+b^ adjusted for child PGS; ^a+b+c^ adjusted for socioeconomic (maternal educational attainment, family size) and maternal (age, early parenthood) characteristics; ^a+b+c+d^ further adjusted for parental conflict. PGS: Polygenic Score for Neuroticism, CI: confidence interval.

There was some evidence for specific indirect effects through maternal worries about feeding ( *B* =0.019, 95%CI 0.003, 0.035, p=0.010) and maternal perceptions and responses to crying in the fully adjusted models ( *B* =0.018, 95%CI 0.004, 0.032, p=0.012; Table 5). Sensitivity analyses using bias-corrected (BC) bootstrapping (n=1,000; MacKinnon *et al.*, 2004) to estimate indirect effects led to similar conclusions, albeit with higher *p*-values (Table S1, Extended data ( Culpin *et al*., 2021)). 

It was only possible to model the EPDS as a sum-score in imputed data analyses due to rare values on specific individual items. Thus, to investigate the impact of missing data we compared equivalent models in complete (n=5,881) and imputed (n=7,523) data sets using the EPDS as a sum-score. The results from the analyses with imputed data ( Table 6; further details in Results S1, Extended data ( Culpin *et al*., 2021)) supported our findings: the total indirect effects were in the same direction and led to the same overarching conclusions as in the complete case analyses using EPDS as a sum-score ( Table 7).

**Table 6.** Estimates of the direct and mediated effects in the specific factors mediator model unadjusted and adjusted for child PGS and antenatal confounders in imputed sample (n=7,523; exposure: maternal depression modelled as a sum-score).

	Model estimates (n=7,523)							
Effect Size ^1^	Unadjusted model ^a^		Adjusted model ^a+b^		Adjusted model ^a+b+c^		Adjusted model ^a+b+c+d^	
	*Β* [95% CI]	P- value	*Β* [95% CI]	P- value	*Β* [95% CI]	P- value	*Β* [95% CI]	P- value
*1. Total effect* Early maternal postnatal depression on offspring depression	0.020 [0.006, 0.034]	0.003	0.020 [0.006, 0.034]	0.003	0.013 [-0.001, 0.027]	0.064	0.012 [-0.002, 0.026]	0.076
*2. Direct effect* Early maternal postnatal depression on offspring depression, accounting for all specific parenting factors	0.013 [-0.001, 0.027]	0.065	0.013 [-0.001, 0.027]	0.064	0.007 [-0.007, 0.021]	0.315	0.007 [-0.007, 0.021]	0.336
*3. Total indirect* Early maternal postnatal depression on offspring depression, through all specific parenting factors	0.007 [0.001, 0.013]	0.012	0.007 [0.001, 0.012]	0.013	0.006 [0.001, 0.009]	0.027	0.005 [0.001, 0.009]	0.026
*4. Specific indirect effect* ^2^ Early maternal postnatal depression on offspring depression, through:								
*Worries about feeding*	0.003 [0.001, 0.005]	0.015	0.003 [0.001, 0.005]	0.016	0.003 [0.001, 0.005]	0.016	0.003 [0.001, 0.005]	0.017
*Perceptions and responses to * *crying*	0.001 [-0.001, 0.003]	0.253	0.001 [-0.001, 0.003]	0.253	0.001 [-0.001, 0.003]	0.086	0.001 [-0.001, 0.003]	0.078

*Note:* ^1^ Effect size are unadjusted and adjusted probit regression coefficients ( *B* unstandardised); ^2 ^to reduce table complexity, only results for specific indirect effect through maternal worries about feeding and perceptions and responses to crying are presented; ^a^ unadjusted model; ^a+b^ adjusted for child PGS (direct pathway between maternal and offspring depression only); ^a+b+c^ adjusted for socioeconomic (maternal educational attainment, family size) and maternal (age, early parenthood) characteristics; ^a+b+c+d^ further adjusted for parental conflict. PGS: Polygenic Score for Neuroticism, CI: confidence interval.

**Table 7.** Estimates of the direct and mediated effects in the specific factors mediator model unadjusted and adjusted for child PGS and antenatal confounders in complete sample (n=5,881; exposure: maternal depression modelled as a sum-score).

	Model estimates (n=5,881)							
Effect Size ^1^	Unadjusted model ^a^		Adjusted model ^a+b^		Adjusted model ^a+b+c^		Adjusted model ^a+b+c+d^	
	*Β* [95% CI]	P- value	*Β* [95% CI]	P- value	*Β* [95% CI]	P- value	*Β* [95% CI]	P- value
*1. Total effect* Early maternal postnatal depression on offspring depression	0.016 [0.001, 0.032]	0.047	0.016 [0.001, 0.032]	0.044	0.010 [-0.006, 0.026]	0.218	0.009 [-0.007, 0.025]	0.257
*2. Direct effect* Early maternal postnatal depression on offspring depression, accounting for all specific parenting factors	0.009 [-0.009, 0.027]	0.306	0.009 [-0.009, 0.027]	0.287	0.004 [-0.013, 0.022]	0.678	0.003 [-0.015, 0.021]	0.736
*3. Total indirect* Early maternal postnatal depression on offspring depression, through all specific parenting factors	0.007 [-0.001, 0.015]	0.088	0.007 [-0.001, 0.015]	0.044	0.006 [-0.002, 0.014]	0.080	0.006 [-0.002, 0.014]	0.071
*4. Specific indirect effect* ^2^ Early maternal postnatal depression on offspring depression, through:								
*Worries about feeding*	0.004 [0.001, 0.008]	0.009	0.004 [0.001, 0.008]	0.010	0.004 [0.001, 0.008]	0.011	0.004 [0.001, 0.008]	0.012
*Perceptions and responses to* *crying*	0.001 [-0.001, 0.003]	0.073	0.001 [-0.001, 0.003]	0.074	0.002 [0.001, 0.004]	0.030	0.002 [0.001, 0.004]	0.027

*Note:* ^1^ Effect size are unadjusted and adjusted probit regression coefficients ( *B* unstandardised); ^2^ to reduce table complexity, only results for specific indirect effect through maternal worries about feeding and perceptions and responses to crying are presented; ^a^ unadjusted model; ^a+b^ adjusted for child neuroticism score (direct pathway between maternal and offspring depression only); ^a+b+c^ adjusted for socioeconomic (maternal educational attainment, family size) and maternal (age, early parenthood) characteristics; ^a+b+c+d^ further adjusted for parental conflict. PGS: Polygenic Score for Neuroticism.

However, stronger evidence for the total indirect effect emerged in the imputed data analyses ( Table 6; unadjusted model: *B*=0.007, 95%CI 0.001, 0.013, p=0.012; fully adjusted model: *B*=0.005, 95%CI 0.001, 0.009, p=0.026) compared to complete case analyses ( Table 7: unadjusted model: *B*=0.007, 95%CI -0.001, 0.015, p=0.088; fully adjusted model: *B*=0.006, 95%CI -0.002, 0.015, p=0.071). The sensitivity analyses with diagnosis of depression (CIS-R; Lewis *et al*., 1992) and depressive symptoms (MFQ; Messer *et al*., 1995) at 18 years resulted in the same pattern of results, with stronger evidence for the direct effect in the fully adjusted model with depressive symptoms as an outcome (Results S1 and Table S2, Extended data ( Culpin *et al*., 2021)).

## Discussion

### Main findings

In this population-based cohort study, we found some evidence for an association between maternal PND and increased risk of offspring depression at age 24 years, which was explained by maternal nurturing behaviours during early childhood. Maternal PND was associated with fewer maternal nurturing behaviours, which, in turn, were associated with increased risk of offspring depression. Indeed, in the fully adjusted model, there was evidence of an indirect pathway through maternal nurturing behaviours (albeit with wide 95% CIs), but no evidence of a remaining direct association between maternal and offspring depression. The indirect pathway was driven by two specific parenting factors, maternal worries about feeding and perceptions and responses to crying, although the effect sizes were notably small.

Our findings are consistent with previous longitudinal research linking parental depression with less optimal parenting behaviour ( Suveg *et al*., 2011), which in turn increases the risk for offspring depression ( Caron *et al*., 2006; Johnson *et al*., 2001). Bifulco *et al*. (2002) found that maternal depression exerted no direct effect on offspring psychopathology once a mediating pathway through a composite measure of offspring neglect and abuse was accounted for. However, these studies focused on harsh dimensions of parenting ( Bifulco *et al*., 2002; Johnson *et al*., 2001), with less emphasis on day-to-day parenting strategies geared to meet and attend to children’s basic nurturing needs. This study extends existing literature with evidence that variance in maternal nurturing behaviours may constitute an important explanatory mechanism of the association between maternal and offspring depression. The negative emotions that characterise maternal depression may be colouring the experience of day-to-day parenting and maternal responses to feeding and crying ( Berg-Nielsen *et al.*, 2002).

We found evidence of specificity for two parenting factors in mediating the mother-to-child depression effect, namely maternal worries about feeding and perceptions and responses to crying. Items around worries about feeding and crying were self-reported, which may not reflect actual maternal behaviours. However, what is shared between these two factors is emotional concern, which may be a critical component for the mediating mechanisms in the association between maternal and offspring depression. Arguably, measurement of parenting practices that reflects feelings, beliefs and perceptions may be better captured by parent-reports than independently observed behaviours ( Smith, 2011). Mothers who experience depression are more prone to emotional dysregulation ( Goodman & Gotlib, 1999), which may translate into inconsistent or harsh parenting. Parent and child co-regulate emotions in infancy ( Calkins, 2011); thus, if the mother is feeling negative emotions when dealing with basic nurturing needs, the infant may also experience negative emotions. Consistent responsiveness to the infants’ needs also provides a predictable scaffolding, which empowers the infant to feel in control of their environment ( Crittenden & Landini, 2015). If consistent responsiveness is disrupted, children’s self-regulatory competence may also be affected potentially contributing to long-term depression.

### Strengths and limitations

An important issue in the context of parenting is the consistency and stability of parenting behaviours. It is possible to assume that maternal early nurturing behaviours continue to exert effects on parenting practices across the lifespan, influencing offspring depression in early adulthood ( Kerr & Capaldi, 2019). Further research is needed to address continuity, stability and change of parenting behaviours and their roles in the intergenerational transmission of depression.

Associations between parenting and infant feeding, crying and sleeping are complex and, most likely, bidirectional ( Avinun & Knafo, 2014; Mills-Koonce *et al*., 2007). Our findings suggest that maternal PND is associated with maternal nurturing behaviours in early childhood. In line with transactional developmental models, infants with more difficult feeding, crying and sleeping patterns may also influence maternal PND ( Murray *et al*., 1996). Examination of possible bidirectionality was outside the scope of the current study, but we accounted for possible evocative child effects and shared genetic liability for depression in mothers and offspring and related phenotypes, such as parenting experiences, by including genetic liability scores for neuroticism ( Knafo & Jaffee, 2013). It should be noted that we only included genetic scores for neuroticism, which explained a small proportion of the variance in the outcome; thus, shared genetic variance in depression and parenting not captured by such scores is likely to play a role in the associations between PND, parenting and offspring depression, precluding causal interpretation.

The strengths of the study include a longitudinal design and a large community-based sample that enabled us to examine the long-term association between maternal PND and offspring depression in early adulthood, as well as elucidate possible transmission pathways. To our knowledge, no previous studies have examined maternal nurturing behaviours as possible explanatory mechanisms in the association between maternal and offspring depression. Furthermore, we utilised clinical diagnosis of offspring depression and accounted for a range of confounders, including child neuroticism PGS. Modelling basic maternal nurturing behaviours concerning feeding, crying and sleeping as a latent factor also enabled us to capture maternal behaviours across early childhood (birth to 3.5 years).

A limitation of the study relates to sample attrition, which is similar to that observed in other population-based studies ( Boyd *et al*., 2013; Fraser *et al*., 2013). Sample attrition may have implications for internal validity, given that participants from lower socio-economic background and those with depression were under-represented in our complete sample. We addressed bias associated with selective attrition by controlling for factors known to predict missingness and by imputing missing data in our exposure, outcome and confounders. The results from the analyses with imputed data supported our findings with the total indirect effects being in the same direction compared to complete case analyses.

Non-independence of measurement and reporting bias, whereby maternal depression and parenting practices are reported by the same informant (the mother), is another limitation. Evidence suggests that depressed mothers may report more negative parenting ( Burt *et al*., 2005), potentially biasing the indirect effects to the null and, therefore, over-estimating the direct effects. Arguably, reports of specific behaviours assessed using relatively neutral/functional items (e.g., ‘frequency child made to go to bed’) may be less susceptible to bias than global assessments of parenting style ( Goodman & Gotlib, 1999). Even though maternal depression may influence reports of perceived worries surrounding parenting, the offspring depression outcome in our study was child-reported (i.e., no shared bias), suggesting that at a minimum, maternal reports of their parenting are more predictive of offspring outcomes than reports of maternal depression itself. Maternal depression in pregnancy and throughout childhood as a possible alternative mechanism should also be noted. The effect size of the association between maternal PND and offspring depression (direct effect) in our sample was small, suggesting that the accrued effects of exposure to chronic maternal depression across the child’s life may be of importance. Examination of timing and chronicity of maternal PND was not the focus of this paper and has been extensively explored in relation to offspring outcomes in ALSPAC ( Netsi *et al*., 2018) and other population-based studies ( Hammen & Brennan, 2003). Better powered studies are also needed to examine possible sex differences in maternal nurturing behaviours and their mediating role in the association between maternal PND and offspring depression.

Our measure of parenting was self-reported rather than independently assessed, potentially biasing the estimation of associations between some parenting behaviours and offspring depression ( Kendler & Baker, 2007). However, we modelled parenting items across several time points, arguably capturing a more comprehensive picture of maternal nurturing behaviours compared to a one-off assessment. Although we adjusted for a range of possible measured confounders, residual confounding remains a possibility. The sizes of the associations were relatively small; however, in this study we looked at variance in maternal depression and parenting in the *whole* population sample where even small effects can have a meaningful impact ( Bornstein, 2014). Finally, we did not explore measures of the fathers’ parenting behaviour, which are known to be important ( Parke & Cookston, 2019).

### Implications of the research

Our findings indicate that maternal nurturing behaviours may play an important role in the association between maternal PND and offspring depression in adulthood, even if indirect effect sizes were small. Further studies to examine whether these associations are causal are needed to strengthen these findings. If they are causal, interventions that identify and treat depression early, as well as enhance maternal nurturing behaviours concerning feeding, crying and sleeping to address worries and emotional reactivity around such activities, may contribute to reducing intergenerational transmission of mental health risk. This therapeutic strategy may be particularly important in light of evidence suggesting that parenting interventions focused on active acquisition of parenting skills and increased parenting confidence are effective in improving offspring development ( Kaminski *et al*., 2008). Future research should also focus on examining patterns of complex and cascading processes associated with maternal PND and less optimal parenting, which may contribute to adverse offspring outcomes, including depression. It is possible that parenting difficulties in early infancy set in motion processes that contribute to increased risk of offspring depression overtime. Future research should focus on disentangling such complex processes to improve offspring outcomes and to identify probable effective targets for interventions.

## Data Availability

ALSPAC data are available through a system of managed open access. The study website contains details of all the data that is available through a fully searchable data dictionary and variable search tool data dictionary. The application steps for ALSPAC data access are highlighted below. 1. Please read the ALSPAC access policy, which describes the process of accessing the data in detail, and outlines the costs associated with doing so. 2. You may also find it useful to browse the fully searchable research proposals database, which lists all research projects that have been approved since April 2011. 3. Please submit your research proposal for consideration by the ALSPAC Executive Committee. You will receive a response within 10 working days to advise you whether your proposal has been approved. If you have any questions about accessing data, please email alspac-data@bristol.ac.uk. Open Science Framework: Maternal postnatal depression and offspring depression at age 24 years in a UK-birth cohort: the mediating role of maternal nurturing behaviours concerning feeding, crying and sleeping. DOI: https://doi.org/10.17605/OSF.IO/RFESM ( Culpin *et al*., 2021). This project contains the following extended data: Supplementary_Maternal Nurturing.docx (Supplementary methods and results) Data are available under the terms of the Creative Commons Zero "No rights reserved" data waiver (CC0 1.0 Public domain dedication).
