# Supplementary material for: Maternal postnatal depression and offspring depression at age 24 years in a UK-birth cohort: the mediating role of maternal nurturing behaviours concerning feeding, crying and sleeping
Source: Wellcome Open Res. 2022 Oct 19;6:187. Originally published 2021 Jul 22. [Version 2] doi: 10.12688/wellcomeopenres.17006.2 (PMC9585356; doi:10.12688/wellcomeopenres.17006.2)
Supplement: Peer review history

# Peer review: Reviewer response for version 2

**DOI:** 10.21956/wellcomeopenres.20472.r52926

**Published:** 2022-10-20

**Authors:** Vivette Glover

**Affiliations:** 1 Institute of Reproductive and Developmental Biology, Imperial College London, London, UK

I approve this version.

Is the work clearly and accurately presented and does it cite the current literature?

Yes

If applicable, is the statistical analysis and its interpretation appropriate?

I cannot comment. A qualified statistician is required.

Are all the source data underlying the results available to ensure full reproducibility?

Yes

Is the study design appropriate and is the work technically sound?

Yes

Are the conclusions drawn adequately supported by the results?

Partly

Are sufficient details of methods and analysis provided to allow replication by others?

Yes

Reviewer Expertise:

Perinatal psychobiology. Not an expert in statistics.

I confirm that I have read this submission and believe that I have an appropriate level of expertise to confirm that it is of an acceptable scientific standard.

# Peer review: Reviewer response for version 1

**DOI:** 10.21956/wellcomeopenres.18778.r49736

**Published:** 2022-07-18

**Authors:** E. Mark Cummings

**Affiliations:** 1 Department of Psychology, University of Notre Dame, South Bend, IN, USA

This manuscript presents a timely and important analysis of relations between maternal postnatal depression and offspring depression in early adulthood (age 24).  Strong points include the large sample size based on a UK-based birth cohort study, the Avon Longitudinal Study of Parents and Children (ALSPAC), the long-term prospective longitudinal research design, and the analysis of possible mediating effects of maternal basic nurturing parenting behaviors including worries about feeding and response to crying. 

 Analyses supported that adverse outcomes of maternal postnatal depression on offspring depression were mediated by early parenting behaviors.  

 The manuscript makes a very important contribution by calling attention to the point that maternal post-natal depression does not necessarily directly lead to long-term problems in youth as a direct effect but more likely leads to later depression or other problems by setting in motion family processes over time that increase consequent risk. In this instance, the data support that early parenting behaviors are a link in a chain of consequent causal processes that indirectly leads to later risk for offspring adult depression. 

 A future direction that the authors may want to encourage is more study of how maternal post-natal depression and consequent parenting problems may result in a pattern of multiple and possible complex cascading processes over time that contribute to increased risk for offspring adult depression.  That is, it seems unlikely that early parenting problems directly lead to later adult depression but instead early parenting problems may set in motion other (at this point unknown) processes that contribute to heightened risk over time.   

 With regard to the manuscript, the authors may want to reconsider which figures and tables to include in the text or, alternatively, in the supplemental materials.  For example, the inclusion of Figure S2, Tables S1 and S4, and Results S1 seem like they may be informative but some of the current materials (e.g., Figures 1 and 2) did not seem especially informative.  I found myself a little frustrated at times when I could not find figures or tables in the text that seemingly would provide valuable information.

 The manuscript would also benefit from the addition of an “analysis plan” paragraph that briefly outlines the plan for analyses and perhaps the rationale for the analysis plan that is followed.  The text presents the results of many analyses, including total effects, total indirect effects, direct effects, imputed analyses, adjusted and unadjusted models, sensitivity analyses, and other analysis directions, with sometimes seemingly contradictory results (e.g., a significant “total effect” – is that evidence for a significant direct effect?).  I suspect all of the analyses presented make perfect sense but readers may benefit from a further explication of the analysis plan.

Is the work clearly and accurately presented and does it cite the current literature?

Yes

If applicable, is the statistical analysis and its interpretation appropriate?

Yes

Are all the source data underlying the results available to ensure full reproducibility?

Yes

Is the study design appropriate and is the work technically sound?

Yes

Are the conclusions drawn adequately supported by the results?

Yes

Are sufficient details of methods and analysis provided to allow replication by others?

Yes

Reviewer Expertise:

Psychology

I confirm that I have read this submission and believe that I have an appropriate level of expertise to confirm that it is of an acceptable scientific standard.

# Peer review: Reviewer response for version 1

**DOI:** 10.21956/wellcomeopenres.18778.r45124

**Published:** 2021-08-10

**Authors:** Vivette Glover

**Affiliations:** 1 Institute of Reproductive and Developmental Biology, Imperial College London, London, UK

This is a careful analysis of the association between maternal postnatal depression and offspring depression at age 24. They use the large ALSPAC cohort. The authors examine the possible mediating effects of early maternal nurturing behaviour and find effects via feeding and response to crying. They suggest that this implies early intervention targeting these aspects of maternal behaviour may be beneficial.

 It remains possible that these early nurturing behaviours are associated with parenting behaviour for the next 20 or more years, and these play a large part in the later offspring depression. This possibility is not discussed in the Discussion and should be. 

 It would be of interest to show the unadjusted risk of the offspring depression if the mother has an EPDS score of >/ 13. The authors say the effects they detect are small, but it would be of interest to also have some idea of the % of the variance of offspring depression they think may be mediated by the mother's nurturing behaviour.

 Figures 1 and 2 do not add much. They could be included in the Supplementary material, and their information covered in the text. It would be of more interest to include tables S2 and S3 in the main paper.

Is the work clearly and accurately presented and does it cite the current literature?

Yes

If applicable, is the statistical analysis and its interpretation appropriate?

I cannot comment. A qualified statistician is required.

Are all the source data underlying the results available to ensure full reproducibility?

Yes

Is the study design appropriate and is the work technically sound?

Yes

Are the conclusions drawn adequately supported by the results?

Partly

Are sufficient details of methods and analysis provided to allow replication by others?

Yes

Reviewer Expertise:

Perinatal psychobiology. Not an expert in statistics.

I confirm that I have read this submission and believe that I have an appropriate level of expertise to confirm that it is of an acceptable scientific standard, however I have significant reservations, as outlined above.
